# A case of diffuse pulmonary ossification

**DOI:** 10.1002/rcr2.812

**Published:** 2021-07-28

**Authors:** Makiko Yomota, Tina Kamei, Kie Mirokuji, Tsunekazu Hishima, Yukio Hosomi

**Affiliations:** ^1^ Department of Respiratory Medicine Tokyo Metropolitan Cancer and Infectious Diseases Center Komagome Hospital Tokyo Japan; ^2^ Department of Pathology Tokyo Metropolitan Cancer and Infectious Diseases Center Komagome Hospital Tokyo Japan

**Keywords:** Bronchoscopy, pulmonary ossification, rare pulmonary disease, transbronchial lung biopsy, transplantation

## Abstract

Diffuse pulmonary ossification (DPO) is a rare condition characterized by the formation of bone tissues in the lung. DPO is considered to be accompanied by chronic lung diseases, such as idiopathic interstitial pneumonitis or chronic obstructive pulmonary disease, acute respiratory distress syndrome, or inhalation‐related lung diseases. Most reported cases of DPO were diagnosed during autopsies or surgical specimen. We report a case of DPO after kidney transplantation diagnosed by transbronchial lung biopsy.

## Graphical abstract

Diffuse pulmonary ossification (DPO) is a rare condition characterized by the formation of bone tissues in the lung. Most cases of DPO are diagnosed during autopsies or surgical specimen. We report a case of DPO after kidney transplantation diagnosed by transbronchial lung biopsy.

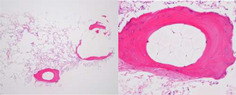

## Introduction

Diffuse pulmonary ossification (DPO) is a rare condition characterized by the formation of bone tissues in the lung. It often occurs in the context of a pre‐existing pulmonary or cardiac disorder, and most cases have been found during autopsy or in surgical specimens [1].

Herein, we report a case of DPO diagnosed by transbronchial lung biopsy (TBLB).

## Case Report

**Figure 1:**
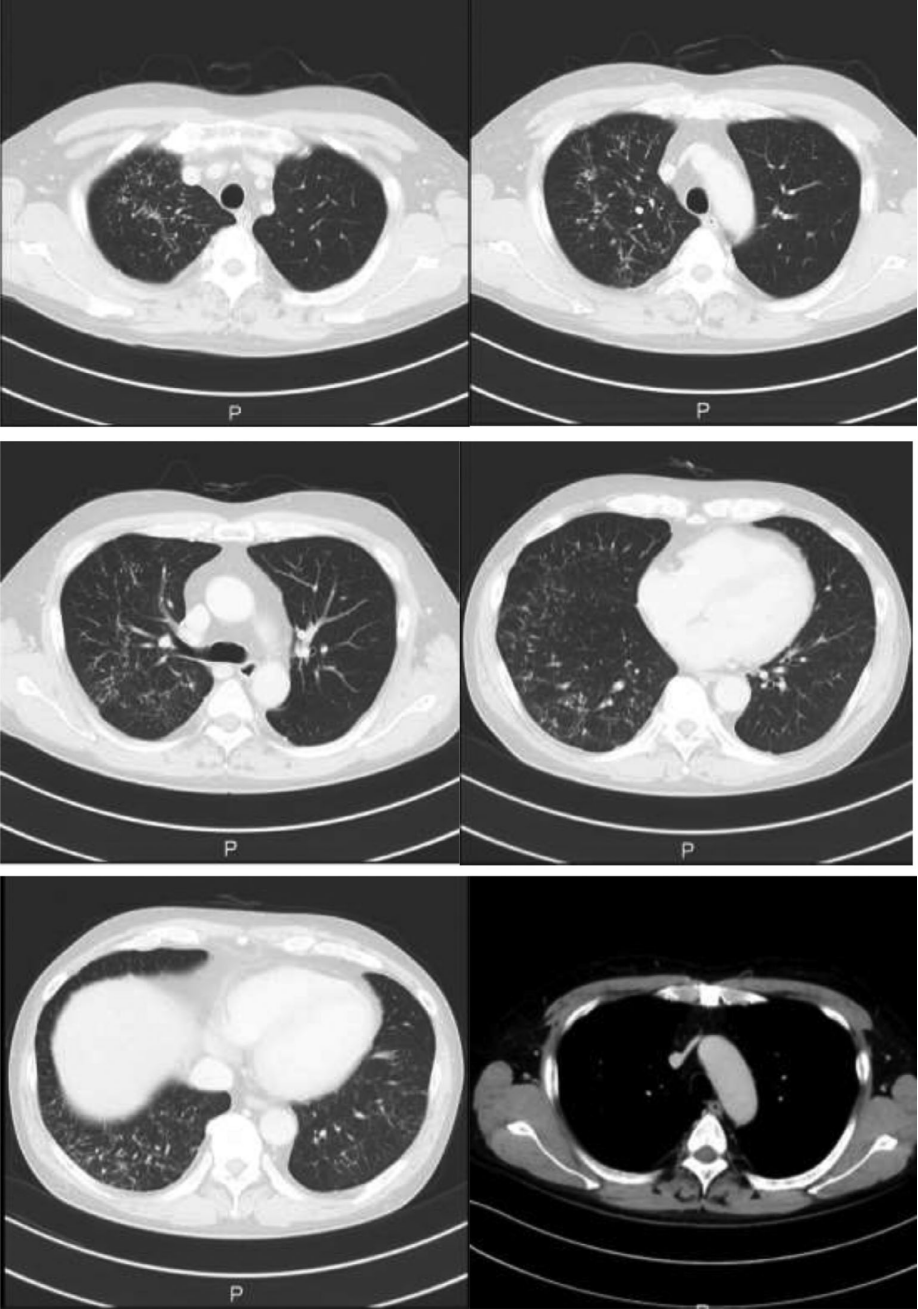
Computed tomography (CT) images demonstrated diffuse irregular nodules bilaterally.

A 50‐year‐old man with early‐stage gastric cancer at the Department of Gastroenterology of our hospital was referred to our department due to a chest X‐ray abnormality. Computed tomography (CT) images demonstrated irregular nodules bilaterally (Fig. 1).

The patient was asymptomatic. Similar chest X‐ray abnormalities had been found at the medical check‐up two years before presentation. He had a 20‐year smoking history. His renal function declined at 30 years due to chronic nephritis, and he underwent artificial haemodialysis for three years. Then, he had a kidney transplant from a living donor at 33 years. His renal function improved and remained stable after kidney transplantation. His medications included 5 mg of prednisolone, 25 μg of cyclosporine, 1 mg of mizoribine, 25 mg of losartan, 50 mg of allopurinol, and 10 mg of nifedipine. He did not have any parathyroid dysfunction and did not take calcium supplements.

His vital signs were normal, with oxyhaemoglobin saturation of 98% in room air. On physical examination, respiratory sounds were normal without any abnormal findings detected. Laboratory tests showed normal values for complete blood counts, serum calcium, liver enzyme, and autoantibody levels, and mild elevation of serum creatinine (1.37 mg/dL). Serum calcium (9.9 mg/dL) and C‐reactive protein (0.3 mg/dL) levels were also normal.

**Figure 2:**
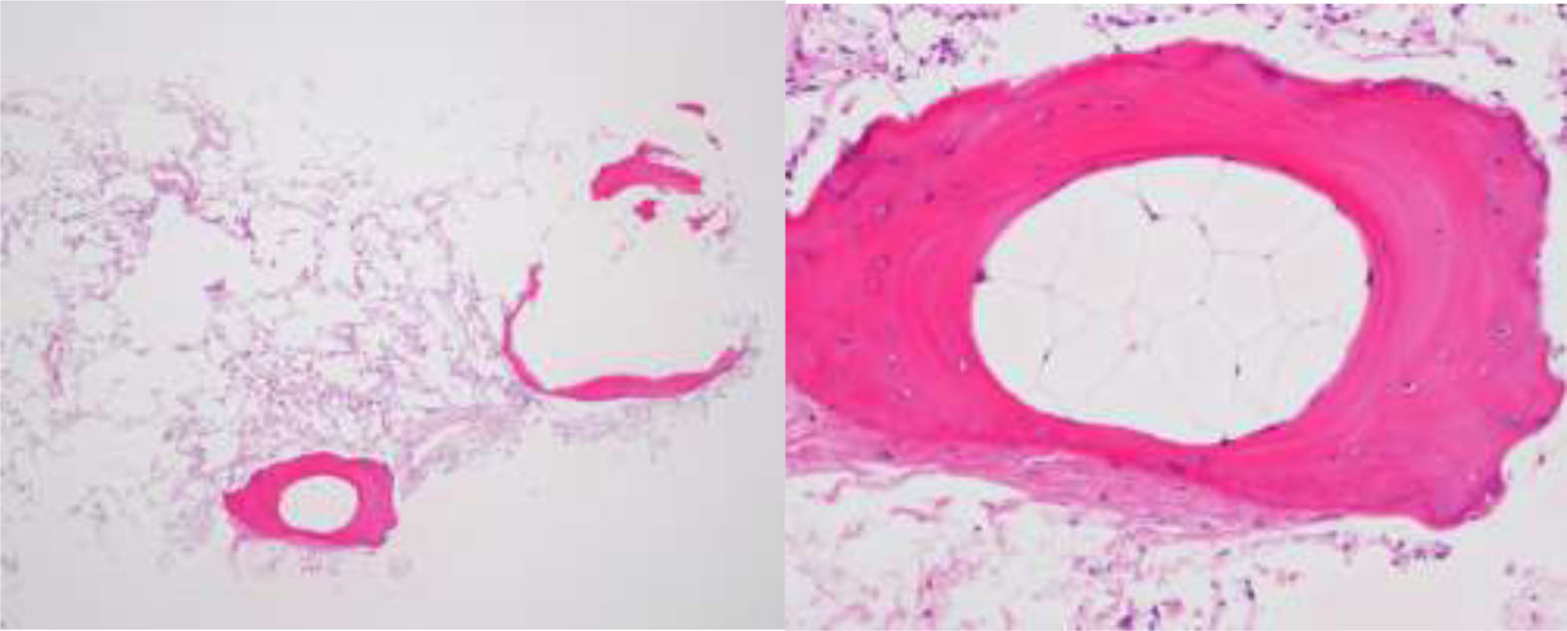
Left: Haematoxylin and eosin stain, low power. Right: High power. This specimen show ossification of alveolar spaces accompanied with marrow elements and fat.

Pulmonary function test results were normal. A diagnostic TBLB was obtained. The specimen showed dendriform ossification of alveolar spaces accompanied by marrow elements and fats (Fig. 2). The bacterial culture of bronchial lavage showed *Haemophilus influenzae* (2+) and was negative for mycobacteria.

Thus, we diagnosed the patient with DPO. His chest X‐ray and pulmonary function test results were stable at five years post‐diagnosis.

## Discussion

Based on our literature review, approximately 50 cases of DPO have been reported to date: 20 diagnosed during autopsies and 23 from surgical specimens. Only one case diagnosed using TBLB has been reported [2]. Our case is rare because we diagnosed DPO with TBLB, a procedure rarely resulting in the diagnosis due to its small sample size and failure to pick up marrow elements.

DPO is considered to be accompanied by chronic lung diseases, such as idiopathic interstitial pneumonitis or chronic obstructive pulmonary disease, acute respiratory distress syndrome, or inhalation‐related lung diseases. Approximately 10 cases of idiopathic DPO have been reported, and a majority of them were accidentally detected in asymptomatic individuals [3]. Our patient was stable for five years, and to our knowledge, only one report in the literature mentions slow progression with long‐term follow‐up [4].

The mechanism of bone formation is unclear; however, angiogenesis and transformation of fibroblasts to chondroblasts and osteoblasts may be involved [5]. Histologically, pulmonary ossification is classified as nodular circumscribed type and dendriform type. The nodular type is typically associated with cardiac disorders, such as mitral stenosis. Dendriform ossification is thought to be idiopathic or follows interstitial fibrosis, but it is difficult to define “idiopathic” as there may be underlying undefined lung inflammation with ossification. On a CT scan, 1–5 mm diameter nodules were observed in the peripheral interstitium that formed contiguous linear nodules connecting lobules together, with some high attenuation visible in the mediastinal window [5].

Other differential diagnoses that show diffuse calcification of the lungs are ectopic pulmonary calcification and pulmonary alveolar microlithiasis. The biggest distinction between these diagnoses and DPO is that pulmonary calcification and pulmonary alveolar microlithiasis are usually not accompanied by bone formation. Ectopic pulmonary calcification has been reported as diffuse alveolar foci in patients with chronic renal disease and often occurs due to hypercalcaemia in patients who have undergone dialysis [6]. Pulmonary alveolar microlithiasis is a rare disorder with an unknown aetiology. Radiologically, it is characterized by “sandy” micronodules, ground‐glass opacities, or calcification of the pleural lining and interlobular septa [6].

Our patient had a history of chronic nephritis, kidney transplantation, early‐stage gastric cancer, hypertension, and hyperuricaemia. He had undergone dialysis for only two years before transplantation and had normal serum calcium. DPO has not been reported as a paraneoplastic condition, and his gastric cancer was resectable by submucosal endoscopy; thus, the relevance of gastric cancer is limited. No other potential causes of pulmonary ossification, such as pulmonary fibrosis or pulmonary haemorrhage, were identified. To our knowledge, no other reports of DPO occurring after kidney transplantation have been published, although whether our case was idiopathic or related to kidney transplantation remains unknown.

### Disclosure Statement

Appropriate written informed consent was obtained for publication of this case report and accompanying images.

### Conflict of Interest

Dr Makiko Yomota reports personal fees from Chugai Pharmaceutical Co., Ltd, Ono Pharmaceutical Co., Ltd, AstraZeneca plc, and Taiho Pharmaceutical Co., Ltd., outside the submitted work. Dr Yukio Hosomi reports personal fees from AstraZeneca, Eli Lilly Japan, Taiho Pharmaceutical, Chugai Pharmaceutical, Ono Pharmaceutical, Bristol‐Myers Squibb, Kyowa Kirin, and CSL Behring, outside the submitted work. The rest of the authors have nothing to disclose.

### Author Contribution Statement

Makiko Yomota wrote the first draft. Tina Kamei, Kie Mirokuji, Tsunekazu Hishima, and Yukio Hosomi revised the article for important intellectual content. All authors approved the final version.
